# Monitoring Influenza Epidemics in China with Search Query from Baidu

**DOI:** 10.1371/journal.pone.0064323

**Published:** 2013-05-30

**Authors:** Qingyu Yuan, Elaine O. Nsoesie, Benfu Lv, Geng Peng, Rumi Chunara, John S. Brownstein

**Affiliations:** 1 Management School, University of Chinese Academy of Sciences, Beijing, China; 2 Children's Hospital Informatics Program, Division of Emergency Medicine, Boston Children’s Hospital, Boston, Massachusetts, United States of America; 3 Department of Pediatrics, Harvard Medical School, Boston, Massachusetts, United States of America; 4 Network Dynamics and Simulation Science Laboratory, Virginia Bioinformatics Institute, Virginia Tech, Virginia, United States of America; 5 Department of Epidemiology, Biostatistics and Occupational Health, McGill University, Montreal, Quebec, Canada; University of Hong Kong, Hong Kong

## Abstract

Several approaches have been proposed for near real-time detection and prediction of the spread of influenza. These include search query data for influenza-related terms, which has been explored as a tool for augmenting traditional surveillance methods. In this paper, we present a method that uses Internet search query data from Baidu to model and monitor influenza activity in China. The objectives of the study are to present a comprehensive technique for: (i) keyword selection, (ii) keyword filtering, (iii) index composition and (iv) modeling and detection of influenza activity in China. Sequential time-series for the selected composite keyword index is significantly correlated with Chinese influenza case data. In addition, one-month ahead prediction of influenza cases for the first eight months of 2012 has a mean absolute percent error less than 11%. To our knowledge, this is the first study on the use of search query data from Baidu in conjunction with this approach for estimation of influenza activity in China.

## Introduction

Seasonal influenza epidemics result in an estimated three to five million cases of severe illness and 250,000 to 500,000 deaths worldwide each year [1]. In order to prepare for the next severe pandemic and better control seasonal influenza epidemics, researchers have proposed several approaches to achieve near real-time surveillance of the emergence and spread of influenza. Some novel approaches for rapid disease outbreak detection and surveillance include online surveillance systems utilizing informal sources such as news reports [2], social media data [3]–[16], and search query data [17]–[20]. The idea of using search query data for detecting outbreaks was first introduced in 2006 [17]. Ginsberg et al [18] later discussed how monitoring search queries on Google could be used to detect influenza outbreaks in the United States. Several studies followed, which pointed to the effectiveness and limitations of detecting influenza epidemics using search query data [19], [20]. Although there are limitations, such as the lack of Internet access in some regions of the world and the noise of irrelevant information, Internet search query data is being explored as a low-cost approach to estimating disease activity in near real-time.

Besides influenza surveillance, search query data has also been widely used for research in fields such as, economics and finance. In the same year as the Ginsberg’s publication [18], several studies investigated the usefulness of Google searches for forecasting unemployment in various countries [21]–[25]. Several papers also used search query data to predict consumption [26], [27], house pricing and sales [28], and travel and consumer confidence [27]. Though studies using web search query data have achieved good results in empirical practice, the field is still young and rapidly developing, with room for discussion and improvement.

We introduce a novel method for estimating influenza activity using search query data from Baidu. Data on Internet searches are available on a daily basis, while routine surveillance data from China’s Ministry of Health (MOH) are typically reported with a one to two-weeks lag. The objective is therefore to estimate present influenza activity based on previously observed laboratory surveillance data plus timely search query data before official reports from China’s MOH. Beyond the use of search query data in a new geographic region and the use of a different search engine, this study is an improvement on other research in this area in that, the keyword selection and composition approach presented is more economical in terms of computational resources and cost compared to the original method by Ginsberg et al [18]. Unlike the United States, in China alternative search engines such as Baidu are more widely used than Google. The market share of Google in China is less than 20%, while that for Baidu is more than 80% [29]. The wide use of Baidu in China makes it a more representative search query source for this analysis.

Several methods have been proposed for detecting and predicting trends of influenza epidemics in China [30]–[32]. However, most of these techniques solely use influenza-like-illness (ILI) or influenza case data. In this study, we use a combination of influenza case counts and real-time search query for modeling and detection of current influenza activity. Improving methods for surveillance, modeling, detection and prediction of influenza epidemics in China is extremely important. Two of the three pandemics of the 20^th^ century are thought to have started in China [38], [39]. In addition, the severe acute respiratory syndrome (SARS) of 2002 had its origins in the Guangdong Province of China. Therefore, refining approaches for rapid detection of outbreaks of influenza and other respiratory illnesses in China should benefit global public health.

### Approach

Given data on influenza activity from an official source, the approach in this paper can be summarized as follows: (i) search for keywords or terms which might be related to influenza; (ii) process keywords by eliminating those unrelated to influenza epidemics, those with an interrupted time-series representing search query volume and those not correlated to the influenza epidemic curve; (iii) define weights and composite search index, and (iv) fit regression model using selected keyword index to influenza case data. Whereby, the fitted model uses both the influenza case data and the search index.

## Methods

### Data Sources

#### Official case counts

The counts shown in Table 1 reflect monthly aggregated influenza case counts from March 2009 to August 2012 for China. The data is publicly available on China’s Ministry of Health (MOH) site (http://www.moh.gov.cn/) and typically released 1–2 week after the end of each month. A network of physicians report laboratory confirmed cases to the MOH on a daily basis. However the data is only released to the public at a monthly resolution. The data is solely laboratory confirmed influenza cases and does not include ILI cases. Furthermore, during the 2009 H1N1 pandemic, infections resulting from the new influenza strain were reported separately from cases resulting from circulating seasonal influenza strains in China [40]. The data in this study is solely for seasonal influenza. No ethics committee approval is required to obtain the data since it is publicly available. In addition, only count data is presented, no personal information is revealed, thereby maintaining confidentiality.

**Table 1:** Influenza case data from China’s MOH.

Month	ICD*	Month	ICD	Month	ICD	Month	ICD	Month	ICD
2009–03	8015	2009–12	29977	2010–09	5114	2011–06	3065	2012–03	21625
2009–04	6794	2010–01	10415	2010–10	4121	2011–07	2654	2012–04	10707
2009–05	7769	2010–02	6595	2010–11	5323	2011–08	3243	2012–05	8520
2009–06	7999	2010–03	8488	2010–12	6529	2011–09	4360	2012–06	6195
2009–07	7791	2010–04	6357	2011–01	6072	2011–10	5525	2012–07	6738
2009–08	14548	2010–05	3865	2011–02	5930	2011–11	7055	2012–08	6793
2009–09	43596	2010–06	2642	2011–03	7299	2011–12	11631		
2009–10	25132	2010–07	2627	2011–04	5727	2012–01	10046		
2009–11	43018	2010–08	3588	2011–05	4130	2012–02	17421		

* ICD is the abbreviation for influenza case data.

#### Search query data from baidu

Baidu’s database (http://index.baidu.com/) contains logs of online search query volume submitted from June 2006. However, since the influenza case count data is available from March 2009, we use Baidu’s data from March 2009 to August 2012. Unlike the case data from the Ministry of Health, Baidu’s search query data is available on a daily basis. The data is therefore converted to monthly counts for analysis. User confidentiality is also maintained, since only the combined term frequency data is available. In addition, Baidu releases search query volume for the entire country.

### Keyword Selection and Filtering

Different keywords have different search frequency and can therefore produce diverse modeling outcomes. So keywords are carefully selected to reflect terms most likely associated with influenza epidemics. Note, observations from previous studies such as Ginsberg et al [18], have indicated that more keywords do not necessarily assure better model fit. The marginal contribution of adding terms to a “saturated” model is limited, but costly. Ginsberg et al [18] only selected 45 significant keywords from 50 million. The method of exhaustion employed by Ginsberg et al [18] is computationally expensive and not easily reproducible by researchers with limited resources [27]. In some cases, researchers have solely relied on keywords recommended by Google [23], [24], [26]. Keywords recommended by search engines tend to be comprehensive, but not always relevant to the subject. Therefore, further analysis is required to extract keywords, which are most pertinent to the study.

Keywords used in this study are obtained from the following Chinese website: http://tool.chinaz.com/baidu/words.aspx (hereafter referred to as keyword tool). Keywords suggested by the keyword tool include recommendations from Baidu, and others mined using semantic correlation analysis from portal websites, blogs, and online reports. “Flu” (“流感” in Chinese) is the core keyword in this study. Upon entering “流感” into the keyword tool, we obtain 94 related keywords (Table 2). Although recommended by the keyword tool, some of the 94 keywords are not related to influenza epidemics in China. We therefore filter the keywords as follows: (i) the selected keywords should represent factors that might influence the influenza epidemic. (ii) The search query data for each keyword should be represented as a sequential time series with a daily, weekly or monthly resolution. (iii) Lastly, the time series of selected keywords should have a maximum cross-correlation coefficient of at least 0.4 with the influenza case data.

**Table 2:** Complete keyword list.

流感(flu)	新流感(new flu)	h1n1流感(h1n1 flu)	本山快乐营猪流感**(Benshan Happy camp swine flu)**	流感吃什么药(influenza drugs)	甲型流感疫苗(type a influenza vaccine)	*甲流感预防知识(the knowledge of type a influenza)*
流感疫苗(influenza vaccine)	预防流感知识(knowledge of influenza prevention)	上流感**{up influenza(song)}**	季节性流感疫苗(seasonal influenza vaccine)	**qq流感大盗下载(qq flu game download)**	甲型流感症状(type a flu symptom)	甲型h1n1流感的症状(the symptoms of type a h1n1 flu)
*甲型h1n1流感(type a h1n1 flu)*	关颖 上流感**{Guan Ying up influenza(song)}**	甲型流感(type a flu)	流感概念股**(flu concept stock)**	流感疫苗价格(the price of influenza vaccine)	如何预防猪流感(how to prevent swine flu)	甲型h1n1流感防治(type h1n1 influenza prevention and control)
*山东流感(Shandong flu)*	***a型流感病毒(type a influenza virus)***	*西班牙流感(Spanish flu)*	***香港流感(Hongkong flu)***	如何预防流感(how to prevent flu)	*h1n1流感的症状(h1n1 flu symptom)*	***甲型h1n1流感治疗(type a h1n1 flu therapy)***
流感的预防措施(prevention measures of influenza)	甲型h1n1流感防控(the prevention of h1n1 flu)	甲流感(h1n1 influenza)	**羊流感(goat flu)**	季节性流感(seasonal flu)	***h3n2流感病毒(h3n2 flu virus)***	甲型h1n1流感资料(type a h1n1 flu information)
***流感的预防(prevention of flu)***	甲型h1n1流感预防(prevention of h1n1 flu)	流感预防措施(the prevention measures of influenza)	***预防猪流感(prevent swine flu)***	***北京流感(Beijing influenza)***	***甲型h1n1流感知识(the knowledge of type a h1n1 influenza)***	甲型流感的预防(prevention of type a flu)
流感症状(influenza symptom)	甲型h1n1流感症状(influenza symptom of h1n1)	**情流感(love flu)**	预防甲型h1n1流感(prevention of type a h1n1 flu)	*甲流感预防(prevention of type a influenza)*	**甲型h1n1流感作文(composition of type a h1n1 flu)**	*甲型流感预防知识(prevention knowledge of type a influenza)*
流感病毒(influenza virus)	流感疫情(Flu epidemic)	预防流感(prevent the flu)	*h1n1流感症状(h1n1 influenza symptom)*	流感传播途径(transmission way of flu)	***甲型h3流感(type a h3 flu)***	*流感疫苗不良反应(Influenza vaccine adverse reaction)*
**qq流感大盗(qq influenza game)**	甲型h1n1流感疫苗(influenza vaccine of h1n1)	流感的传播途径(the transmission way of flu)	***甲流感的症状(the symptom of h1n1 flu)***	流感大流行(influenza pandemic)	***甲型流感 症状(type a flu symptoms)***	*流感疫苗接种(influenza vaccinations)*
流感的症状(the influenza symptom)	*甲型h1n1流感(type a h1n1 flu)*	流感疫苗副作用(Influenza vaccine side effects)	甲流感症状(symptom of h1n1 flu)	流感预防(prevent influenza)	预防甲型流感(prevention of type a flu)	*流感最新疫情(Latest outbreak of influenza)*
***流感防治知识(the knowledge of influenza prevention)***	***流感疫苗接种时间(Influenza vaccination time)***	**h1n1流感手抄报(h1n1 flu Shouchao Bao)**	甲型h1n1流感疫情(epidemic situation of type a h1n1 flu )	流感治疗(flu treatment)	*防控甲型h1n1流感(prevention and control type a h1n1 flu)*	人感染猪流感症状(Human infection with swine flu symptoms)
**情流感菌(love flu virus)**	*北京 流感(Beijing flu)*	**上流感 关颖(up influenza(song) Guan Ying)**	*甲型h3n2流感病毒(type a h3n2 flu virus)*	***新型流感(new influenza)***	**狗流感(dog influenza)**	*如何预防甲型流感(How to prevent influenza a)*
*山东猪流感(Shandong swine flu)*	**预防甲型流感手抄报(Shouchao Bao of prevention type a flu)**	***副流感病毒(Para-influenza)***	***甲型流感的症状(the symptom of h1n1 flu)***	a型流感(type a influenza)	甲型h1n1流感病毒(type a h1n1 flu virus)	***流感疫苗有必要打吗(The flu vaccine is necessary to play)***
h1n1流感预防(h1n1 influenza prevention)	*h1n1流感预防知识(h1n1 knowledge of influenza prevention)*	H1n1流感(h1n1 flu)				

Note: Web users use Chinese characters to search in Baidu. Keywords in English are listed to show the corresponding translation of each Chinese character. The keywords in bold are excluded at filtering step (i). The keywords in italics are excluded at filtering step (ii) and keywords in bold and italics are excluded at filtering step (iii).

Keywords that remain after the filtering analysis are considered for inclusion in the composite search index. The goal of search index composition is to build the most correlative and stable indicator for the influenza case data based on the available information. The search index is composed in two steps. First, we define synthetic weights for each of the keywords. Next, we combine the weighted time series for the keywords.

### Search Index Composition

We consider two approaches for defining synthetic weights: the method of systematic assessment and the strength of the correlation coefficient. The method of systematic assessment [34], [35] involves rating the selected indicator according to the principle of prior evaluation and defining the ratings as weights. The method is comprehensive but highly subjective. Alternatively, the correlation coefficient between the influenza epidemic curve and the keyword frequency curve can be used to represent the weight [18], [33]. This approach is usually combined with Analytic Hierarchy Process (AHP) [36] for better performance. However, solely using the correlation coefficient without adjustments appears to be sufficient for this study.

The search index is defined as: 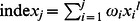, where 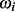 is the weight of the 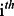 keyword and 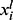 represents the sequence after alignment. Although the definition of the composite index allows for alignment, it is not required for combining the time series in this study since maximum correlations are observed at lag 0. The final set of keywords is selected using the following model:
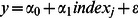
(1)


In (1), 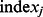 represents the search index for j keywords, y denotes influenza case counts, 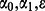 denote the intercept, coefficient and error term respectively.

Using a stepwise approach generally used in the selection of variables in a multiple regression framework, keywords are selected based on their contribution to the model’s goodness of fit. Partial F test is used to evaluate the goodness of fit after adding data for each keyword to the index. A significant F-statistics implies that the keyword should be added to the composite index, and vice versa. The search index is defined based on the model with the best goodness of fit statistics.

The initial model is based on the keyword with the highest correlation with the influenza case data. In this case, “流感预防” (prevent influenza) has the highest correlation at 0.93 at lag 0. Keywords are then added sequentially based on the correlation coefficient and the partial F test is examined for improved fit. The process is repeated until the goodness of fit can no longer be improved.

### Model

As stated, the objective of this paper is to present a method for faster detection of influenza activity in China using search query data. China’s MOH typically releases monthly influenza case data 1–2 weeks into the next month. We therefore aim to provide estimates of case data before the MOH data is publicly available.

The most significant correlations between the composite index and the case data are observed at lag 0 (P  = 0.959) and lag 1 (P = 0.658). Correlations at lags 2 and 3 are 0.491 and 0.227 respectively. We therefore fit the following model:
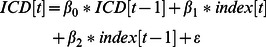
(2)


ICD represents influenza case data, 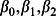 are the coefficients, index is the composite search index and 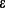
is the error term. The model estimates ICD at time t based on ICD at time t-1 and the composite search index at time t and t-1. For example, case counts for February 2012 are estimated at the end of February based on the composite search index for February and January, and the case count for January. We also examine the residuals to evaluate the adequacy of the model.

The influenza case data is divided into a fitting and validation set. Data from March 2009 to December 2011 is used for model fitting, while data from January 2012 to August 2012 is used for validation. We also consider models with second and third order lags. Models are evaluated based on R-squared, AIC and significance of the coefficients. Studies have suggested that solely using an extrapolation of the influenza activity curve for predictions usually results in a higher error rate [32], [33]. The analysis is performed using the Eviews software.

## Results

**Figure 1:**
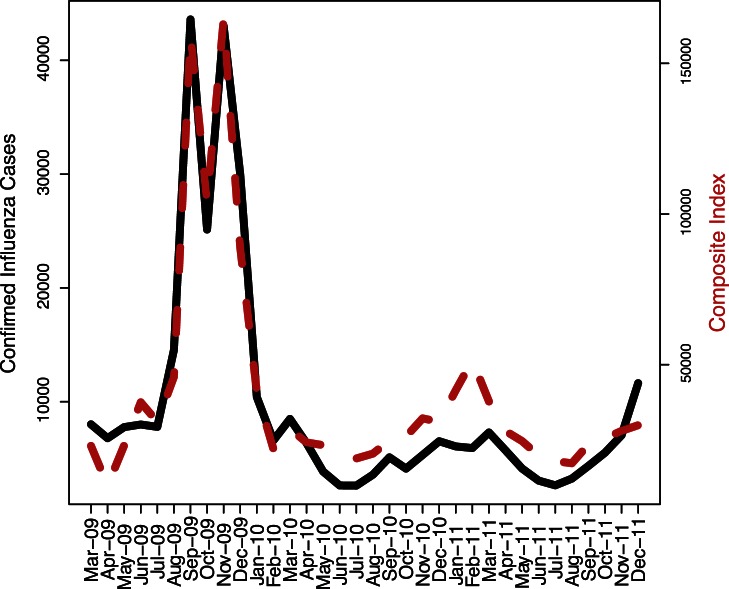
Influenza case data and composite search index.

Based on the filtering analysis, 14 out of the 94 keywords are not related to influenza epidemics, 20 keywords do not have sequential time series due to low search volume and only 40 keywords are significantly correlated to the case data (see Table 2). With the stepwise approach, only 8 of the 40 keywords are used in the composite search index (see Table 3). The estimated cross-correlation coefficient between the search index and influenza case data is 0.96 at lag 0 (Figure 1). Influenza epidemics are observed in the spring and winter as expected. Note that the search index clearly captures the peaks and troughs of the influenza time series curve, thereby making it a good indicator for influenza activity in China.

**Table 3:** Keywords in composite index.

Chinese	流感预防	流感的症状	甲型流感疫苗	流感症状
English	(prevent influenza)	(the influenza symptom)	(type a influenza vaccine)	(flu symptom)
Correlation	0.93	0.92	0.90	0.87
Chinese	流感疫情	流感病毒	流感大流行	a型流感
English	(Flu epidemic)	(influenza virus)	(influenza pandemic)	(type a influenza)
Correlation	0.85	0.63	0.57	0.40

The coefficients 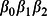 for model (2) are 0.56 (*P = 0.001*), 0.25 (*P<0.001*) and −0.14 (*P = 0.004*) respectively. Note the model’s R-squared is 0.95 and the AIC is 18.50. In addition, the Durbin-Watson test statistic is 1.89 suggesting that autocorrelation is not an issue (see Table 4). The null hypothesis of the Durbin-Watson test is that the autocorrelation parameter is zero.

**Table 4:** Statistical results for model [2].

Variable	Coefficient	Std. Error	t-Statistic	Prob.	R-squared	Durbin-Watson stat
*index*[*t*]	0.253	0.015	17.455	<0.001	0.950	1.887
*index*[*t–*1]	−0.138	0.044	−3.159	0.0036		
*ICD*[*t–*1]	0.555	0.157	3.534	0.0013		
residual	ADF	MacKinnon threshold			Prob*	result
	t-Stat	1%	5%	10%		
	−5.685	−3.654	−2.957	−2.617	<0.001	stationary

Note: ADF is the abbreviation for augmented Dickey-Fuller Test. ICD represents influenza case data.

**Figure 2:**
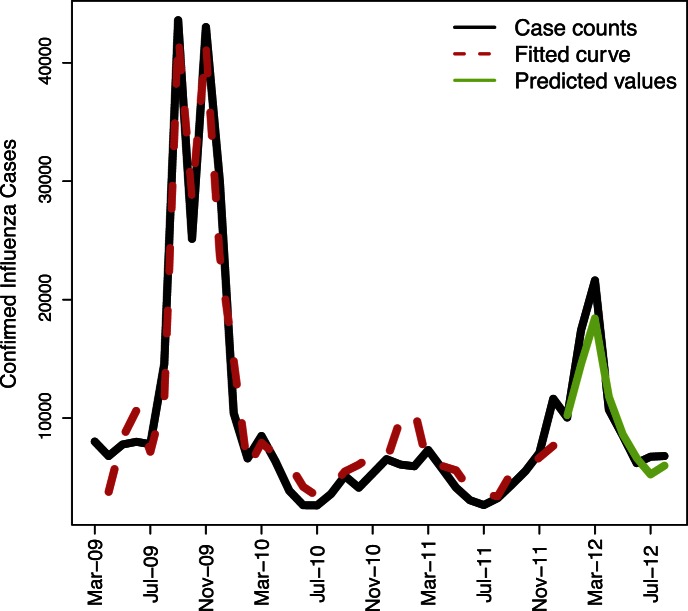
Plot of influenza cases, fitted values and *prediction* based on model [2].

The model is validated by predicting influenza cases one month at a time, from January 2012 to August 2012. The results are listed in Figure 2 and Table 5. The mean absolute percent error of prediction for the consecutive eight months is 10.6% (see Table 5). We also consider models with second order lags and third order lags but neither of their statistical results are better than that of model [2] (see Tables S1 and S2).

**Table 5:** Predicted values and error.

Month	Actual value	Predicted value	Absolute error	Percent absolute error
01–2012	10046	10230	184	1.8%
02–2012	17421	14578	2843	16.3%
03–2012	21625	18429	3196	14.8%
04–2012	10707	11785	1078	10.1%
05–2012	8520	8618	98	1.2%
06–2012	6195	6621	426	6.9%
07–2012	6738	5240	1498	22.2%
08–2012	6793	5983	810	11.9%

## Discussion

We develop a comprehensive method for pre-processing Internet search data for modeling and detecting influenza epidemics in China. The combined keyword index is significantly correlated to the case data and mean absolute percent error of predicting 2012 monthly influenza cases is less than 11% based on one-step predictions for eight months. Although the monthly search query data and influenza case data are almost synchronous, the search query data can still be used in detecting influenza cases because of the time delay of official reports.

This study contributes to the pool of novel sources of data, such as web-based data, used as early indicators for disease outbreaks. To our knowledge, this is the first study utilizing Baidu search query data in conjunction with this approach for estimating influenza activity in China. Baidu has a significantly higher market share than Google in China, thereby making it a better search query source for this study. The proposed approach is not meant to replace actual estimates of influenza cases, rather it is an indicator of influenza activity, which is freely available in near real-time. This is especially relevant for a country such as China, which has been coined the “epicenter of influenza” [39] by some.

However, there are several limitations to using search query data. Although the selected keywords perform well at capturing the temporal trend of the epidemic curve, there is no guarantee that this would be consistent in future dates. Individual behavior is constantly changing and different factors influence keywords queried by individuals. Another limitation is the unavailability of Internet access in rural regions. The China Internet Network Information Center (CNNIC) currently estimates Internet penetration in China at 39.9%. Surveillance using web-query data depends on adequate Internet access. In addition, not all searches on influenza-related terms are necessarily linked to influenza morbidity. Search queries can be a result of panic during a novel respiratory outbreak, coverage of influenza-related deaths in the media, fear or curiosity. Using several years of data in modeling should hopefully mitigate occurrences of panic induced searches since the weight of various keywords is likely to deviate from one influenza season to another. Furthermore, correlation does not imply causation, which suggests that predictions made using such novel data sources should be carefully evaluated.

Limitations also exist in the data used in this study. Influenza-like-illness data might be a better indicator of influenza activity since influenza cases are not always confirmed and case data might underestimate the true burden of the disease. However, China’s Ministry of Health only releases influenza case data for the entire country. In addition, there are likely to be major differences in timing and duration of epidemics from province to province. Analysis at the province level would therefore be more beneficial. Unfortunately, both the case data and search query volume are only available for the entire country. Though, the model can be easily extended to detect influenza activity at a province level.

Although limitations exist, having more methods and resources geared towards infectious disease surveillance provides a step towards rapid detection and control of emerging and re-emerging outbreaks. Public health scientists and epidemiologists could use observations from such approaches as an indicator for further investigations. These tools are freely available in near real-time and can be especially valuable in regions where official reports of case counts are delayed.

## Supporting Information

Table S1 **Results for model with second order lag included.**
(DOCX)

Table S2 **Results for model with second and third order lags included.**
(DOCX)
