# Supplementary material for: Monitoring Influenza Epidemics in China with Search Query from Baidu
Source: PLoS One. 2013 May 30;8(5):e64323. doi: 10.1371/journal.pone.0064323 (PMC3667820; doi:10.1371/journal.pone.0064323)
Supplement: Table S1 — Results for model with second order lag included. (DOCX) [file pone.0064323.s001.docx]

Table S1 Results for model with second order lag included

| **Variable** | **Coefficient** | **Std. Error** | **t-Statistic** | **Prob.** | **R-squared** | **Durbin-Watson stat** |
| --- | --- | --- | --- | --- | --- | --- |
| $index[t]$ | 0.257 | 0.015 | 16.8 | <0.001 | 0.95 | 1.84 |
| $index[t-1]$ | -0.131 | 0.054 | -2.4 | 0.022 |  |  |
| $index[t-2]$ | -0.005 | 0.052 | -0.09 | 0.931 |  |  |
| $ICD[t-1]$ | 0.5411 | 0.196 | 2.75 | 0.011 |  |  |
| $ICD[t-2]$ | -0.010 | 0.194 | -0.051 | 0.960 |  |  |
| residual | ADF | MacKinnon threshold | | | Prob * | result |
|  | t-Stat | 1% | 5% | 10% |  |  |
|  | -5.102 | -3.661 | -2.960 | -2.619 | <0.001 | stationary |
