# Supplementary material for: Monitoring Influenza Epidemics in China with Search Query from Baidu
Source: PLoS One. 2013 May 30;8(5):e64323. doi: 10.1371/journal.pone.0064323 (PMC3667820; doi:10.1371/journal.pone.0064323)
Supplement: Table S2 — Results for model with second and third order lags included. (DOCX) [file pone.0064323.s002.docx]

Table S2 Results for model with second and third order lags included

| **Variable** | **Coefficient** | **Std. Error** | **t-Statistic** | **Prob.** | **R-squared** | **Durbin-Watson stat** |
| --- | --- | --- | --- | --- | --- | --- |
| $index[t]$ | 0.263 | 0.014 | 17.7 | <0.001 | 0.96 | 1.58 |
| $ICD[t-1]$ | 0.577 | 0.189 | 3.0 | 0.006 |  |  |
| $index[t-1]$ | -0.151 | 0.053 | -2.83 | 0.009 |  |  |
| $index[t-2]$ | 0.058 | 0.055 | 1.04 | 0.307 |  |  |
| $index[t-3]$ | -0.095 | 0.048 | -1.96 | 0.062 |  |  |
| $ICD[t-2]$ | -0.295 | 0.208 | -1.46 | 0.170 |  |  |
| $ICD[t-3]$ | 0.441 | 0.179 | 2.45 | 0.022 |  |  |
| residual | ADF | MacKinnon threshold | | | Prob * | result |
|  | t-Stat | 1% | 5% | 10% |  |  |
|  | -4.54 | -3.67 | -2.96 | -2.62 | 0.001 | stationary |
